# Incorporation of visible/near-infrared spectroscopy and machine learning models for indirect assessment of grape ripening indicators

**DOI:** 10.1038/s41598-024-81694-3

**Published:** 2025-04-10

**Authors:** Osama Elsherbiny, Salah El-Hendawy, Salah Elsayed, Abdallah Elshawadfy Elwakeel, Abdullah Alebidi, Xianlu Yue, Wael Mohamed Elmessery, Hoda Galal

**Affiliations:** 1 Agricultural Engineering Department, Faculty of Agriculture, Mansoura University, Mansoura, 35516 Egypt; 2 Department of Plant Production, College of Food and Agriculture Sciences, King Saud University, P.O. Box 2460, 11451 Riyadh, Saudi Arabia; 3 Evaluation of Natural Resources Department, Environmental Studies and Research Institute, University of Sadat City, Menoufia, 32897 Egypt; 4 New Era and Development in Civil Engineering Research Group, Scientific Research Center, Al-Ayen University, Nasiriyah, Thi-Qar 64001 Iraq; 5 Agricultural Engineering Department, Faculty of Agriculture and Natural Resources, Aswan University, Aswan, 81528 Egypt; 6 Institute of Agricultural Resources and Regional Planning, Chinese Academy of Agricultural Sciences, Zhongguancun South Road 12, Haidian District, Beijing, 100081 China; 7 Agricultural Engineering Department, Faculty of Agriculture, Kafrelsheikh University, Kafrelsheikh, 33516 Egypt

**Keywords:** Chemometrics, Decision tree, Fruit quality, Gradient boosting regression, Precision agriculture, Plant sciences, Chemistry

## Abstract

The assessment of grape ripeness is pivotal for optimizing harvest timing and ensuring high-quality fruit production. Traditional methods, relying on manual sampling and chemical analysis, are laborious and expensive. This study proposes an innovative approach combining Visible/Near-Infrared (VIS/NIR) spectroscopy with machine learning techniques—specifically, decision trees (DT) and gradient boosting regression (GBR)—to facilitate a rapid, non-destructive, and cost-effective prediction of key grape ripening indicators such as anthocyanin (An), total acidity (TA), total soluble solids (TSS), and the TSS/TA ratio. The performance of spectral reflectance indices (SRIs) in correlating with these ripening metrics across different grape ripening stages was examined. The study findings revealed notable variations in ripening indicators across stages, with the newly developed SRIs outperforming the existing indices. The application of dual and triple-band SRIs yielded strong correlations with An (R^2^ = 0.75–0.88) and TSS (R^2^ = 0.64–0.76), and moderate correlations with TA (R^2^ = 0.63–0.70), but showed weaker associations with the TSS/TA ratio (R^2^ = 0.15–0.52). Incorporating these SRIs into the DT and GBR models significantly enhanced the accuracy of ripening indicator predictions. The integration of multiple SRIs resulted in the most precise models. The DT model delivered outstanding performance for An (R^2^ = 0.87, RMSE = 87.81) and TSS/TA (R^2^ = 0.74, RMSE = 3.12). Meanwhile, the GBR model excelled in predicting TSS (R^2^ = 0.82, RMSE = 0.92) and TA (R^2^ = 0.70, RMSE = 0.05). Overall, the combination of VIS-NIR spectroscopy and machine learning offers a promising and efficient approach for assessing grape ripeness, providing a practical solution for the agricultural industry.

## Introduction

Table grapes (*Vitis vinifera* L.) are globally cultivated for their rich content of minerals, vitamins, sugars, organic acids, and medicinal properties^1^. The FAO reports that grape cultivation spans 7.5 million hectares worldwide, with an annual production value exceeding $70 billion^2^. Environmental factors significantly impact fruit quality and market prices. Though table grapes come in various varieties, they share the key trait of being non-climacteric, meaning their ripening process stops once harvested. Therefore, determining optimal ripeness is crucial for grape producers, as it affects quality, market value, and sales^3–5^. Harvesting too early lowers quality, while harvesting too late may cause drying and increased microbial decay^6–8^. Key factors for determining harvest timing include geography, climate, grape variety, growing season, and purpose. Ripeness is measured through physiological and biochemical changes, including total soluble solids (TSS), titratable acidity (TA), and their ratio (TSS/TA)^6,8,9^. Grapes with low TSS and high TA are considered immature^4,10,11^, while anthocyanin (An) levels increase in colored varieties as ripening progresses^12,13^.

Traditionally, grape ripeness was assessed using chemical analysis with specialized tools like refractometers and colorimeters, along with vision-based methods for physical characteristics. However, these techniques are challenging due to the need for large, representative samples and skilled operators, making them destructive, costly, and impractical for large-scale use. Consequently, research has focused on developing nondestructive, rapid, low-cost, and real-time alternatives. In recent years, proximal remote sensing has emerged as a promising alternative to traditional methods for assessing grape ripeness and determining optimal harvest timing. These techniques can rapidly collect a range of data on grape maturity across large samples without physical intervention^4,6,14–16^. One such method is portable visible/near-infrared (VIS/NIR) or NIR spectroscopy, which utilizes sensors to detect changes on the fruit’s surface across various wavelengths, offering valuable insights into its maturity.

As grapes ripen, their metabolic changes include alterations in cell wall structure, decreases in chlorophyll and TA, and increases in pigments like carotenoids and An, as well as TSS^4,17,18^. These changes influence the fruit’s spectral reflectance across the electromagnetic spectrum, with notable effects in the VIS range (400–700 nm), the red edge (650–720 nm), and the NIR range (700–1200 nm)^17^. Spectroscopy devices covering these ranges can detect ripening changes. For instance, Fatchurrahman et al.^19^ demonstrated that VIS/NIR (400–1000 nm) and NIR (900–1700 nm) spectroscopy, combined with Partial Least Squares Regression (PLSR), effectively assessed TSS, TA, and phenols in goji berries. The best predictions were in the VIS-NIR region, with R^2^ values of 0.94 for TSS, 0.84 for TA, and 0.62 for phenols. Similarly, Pourdarbani et al.^20^ employed VIS/NIR spectroscopy (400–1000 nm) to predict ripeness in Fuji apples, focusing on firmness, starch content, and TA. Artifical neural network (ANN) outperformed PLSR, achieving the highest correlations, particularly for starch content (0.969) and TA (0.947). Pissard et al.^21^ applied partial least squares (PLS) to predict TSS in apples from NIR spectra, achieving R^2^ values of 0.89 for XDS (X-ray Diffraction Spectrometer) and 0.91 for MicroNIR. Ping et al.^4^ identified an absorption peak between 900–970 nm in grapes, linked to ripening. Using PLSR, they predicted SSC (soluble solids content) and TA with high accuracy (R^2^ = 0.94), highlighting the effectiveness of VIS-NIR spectroscopy for non-destructive grape quality assessment. While these studies show the potential of VIS/NIR and NIR spectroscopy for assessing fruit ripeness, few studies have focused on using portable VIS/NIR devices to predict ripeness-related traits like TSS, TA, and An in table grapes.

Effective wavelengths in the VIS and NIR spectra are used in spectral reflectance indices (SRIs) like the anthocyanin index (NAI), normalized chlorophyll index (NCI), NDVI, PRMI, and greenness index (GI) to track biochemical and biophysical changes during fruit ripening^22–24^. SRIs are particularly useful for monitoring fruit ripeness^25–30^. For instance, Elsayed et al.^26^ found that SRIs were strongly correlated with TSS in orange, guava, and mandarin, with R^2^ values up to 0.87. The two-band SRI_450,640_, from the VIS blue and red regions, showed the highest R^2^ values (0.95 and 0.90) for TSS and firmness in ripening bananas. Further development of optimized SRIs is needed to ensure their reliability in estimating grape ripeness, as existing SRIs often produce inconsistent results across different crops, environments, and spatial conditions. Identifying the most effective algorithms for new SRIs is crucial to accurately assess fruit quality in varied conditions. While previous research has focused mainly on two-band SRIs, this study is significant for identifying the best three-band SRIs using 3-D correlogram maps, enhancing accuracy in fruit quality evaluation. SRIs offer a simple way to assess physical and biochemical characteristics, making them useful for large-scale monitoring and control. However, each SRI is limited to two or three band combinations, which can hinder the development of effective SRIs for evaluating a wide range of fruit traits, especially under variable conditions. This limitation can lead to the omission of key data, making models based on few wavelengths less effective for accurately assessing fruit quality^31^.

Machine learning (ML), a form of artificial intelligence, is increasingly used to extract information from spectral data and identify patterns between reflectance and fruit quality^32–34^. ML’s self-learning ability enables accurate classification and prediction. Feature selection techniques, essential for identifying key features with high discriminative value, are crucial for model performance. These techniques help reduce overfitting, eliminate irrelevant features, and preserve the integrity of the original data^35,36^. Various methods have been explored to reduce data dimensionality and improve prediction accuracy, including decision trees (DT), gradient boosting regression (GBR), and random forests (RF). Recent studies highlight the benefits of combining different methods to enhance hyperspectral prediction accuracy^37,38^. This ensemble approach integrates multiple algorithms and variable selection techniques, showing superior performance compared to single methods^39–41^. Glorfeld^42^ developed a neural network index with back-propagation to identify key factors. Hyperparameter selection is also crucial for improving machine learning (ML) model performance, fairness, and reproducibility^43,44^, playing a key role in refining prediction models^45^.

This study aimed to accomplish the following objectives: (1) to examine fluctuations in ripening cues (An, TSS, TA, and TSS/TA) across various ripening stages; (2) to identify the most effective two- and three-band SRIs for evaluating these ripening indicators through 2D and 3D slice mappings; and (3) to assess the effectiveness of integrating high-level two- and three-band SRIs with DT and GBR models for predicting the ripening indicators.

## Materials and methods

### Description of plant material and stages of maturity

**Figure 1:**
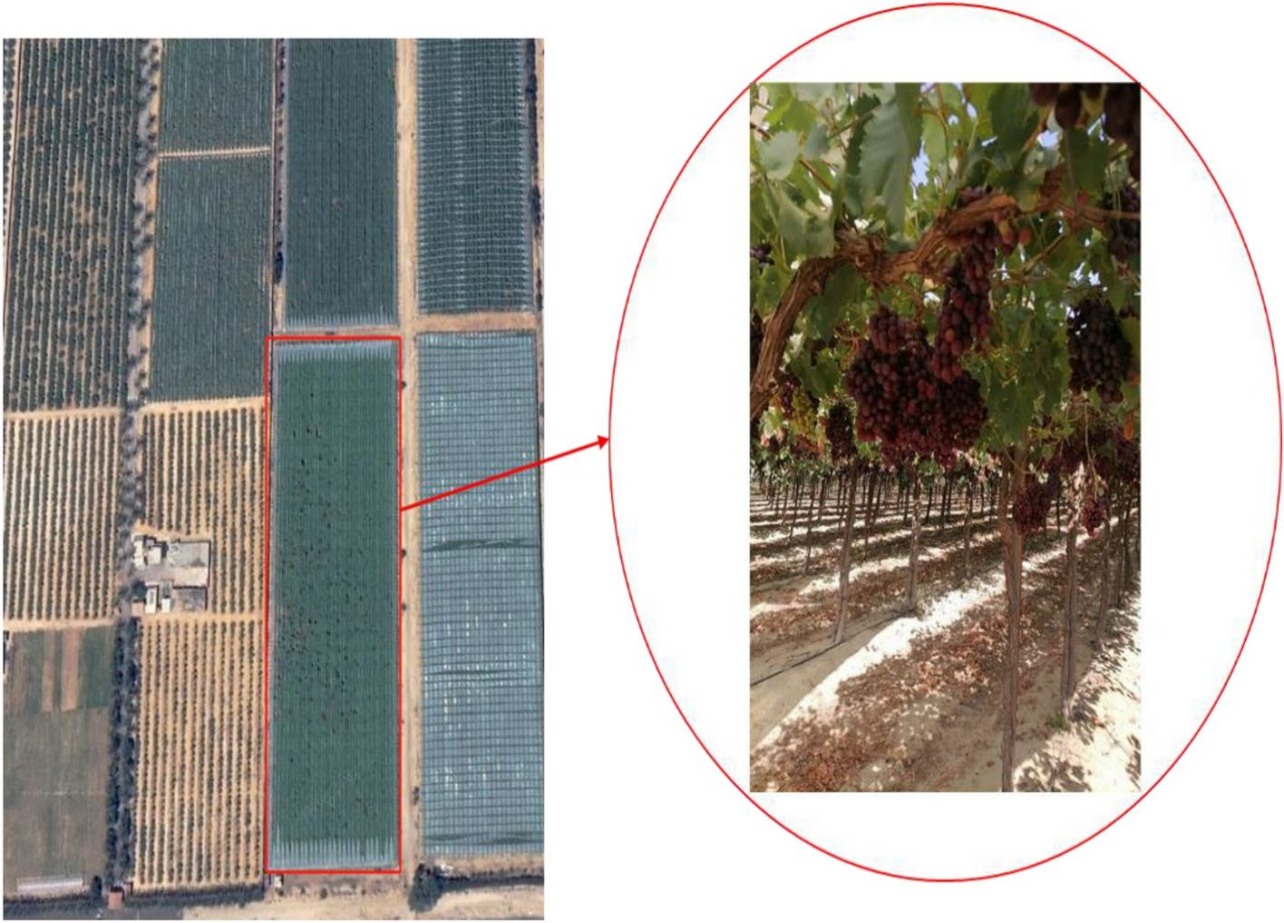
The geographic area of the research region and the collected grape sample.

This work focused on the Flame variety, a popular type of table grapes that is widely cultivated and found across the globe. This variety is an early-growing, seedless type with light red berries. In mid-May 2022, a total of 105 grapevine clusters were collected from a privately owned export orchard and classified into three maturity stages—less mature, semi-mature, and fully mature—each with 35 samples. The orchard is located near the Cairo-Alexandria Desert Road in Nobariya, Beheira Governorate, Egypt (Latitude: 30° 19.11′ N, Longitude: 30° 32.01′ E), as shown in Fig. 1. The clusters were harvested by hand, placed into plastic containers, and promptly transported to the Pomology Laboratory at the Environmental Studies and Research Institute, University of Sadat City, Menoufia, Egypt. In the lab, the clusters obtained were sorted into three groups based on their TSS content. The first group included clusters with TSS ≥ 14%, the second group included clusters with TSS ranging from 14 to 15% (the preferred range for exporting), and the third group included clusters with TSS ≥ 15%. The first, second, and third groups will be referred to as less mature, semi-mature, and fully mature ripened fruits, respectively.

### Observations of spectral reflectance

The spectral reflectance data were collected from each group of ripened fruits using a passive VIS/NIR spectroscopy system (tec5, located in Oberursel, Germany), covering a spectrum range of 302–1148 nm, with a spectral bandwidth of 2 nm, and a viewing angle of 12°. The spectrum data of fruit samples from each group were collected by taking the average of five scans performed at various positions on the cluster. To minimize fluctuations in light exposure, spectral measurements of grape samples were taken within a short timeframe during a sunny period. The spectral reflectance of the grape samples was calibrated using a calibration factor obtained from a white Spectralon ceramic plaque (Labsphere Inc., North Sutton, NH, USA), which has an approximate reflectance of 100%. To minimize background interference, the grape specimens were placed on a black surface, ensuring that the recorded spectral data represented only the reflectance of the grapes. For each measurement, the spectral data were averaged to improve the accuracy of the reflectance values.

### Ripening indicators measurements

Following the acquisition of spectral data from the grape samples, conventional non-destructive methods were employed to evaluate four ripening indicators (An, TSS, TA) across three ripening stages. The grape berries were squeezed to extract the juice, excluding the skin. TSS was measured using a hand-held digital brix refractometer (Atago, Japan), and TA was determined by titrating the samples with 0.1 N NaOH and expressed as a percentage of tartaric acid. The maturation index was calculated by dividing the TSS values by the TA values, and the result was recorded as the TSS/TA ratio. Total anthocyanin content in grape berry skin was measured using the method outlined by Peppi et al.^46^. Briefly, 1g of berry skin was washed with de-ionized water, dried with sterilized tissues, and then placed in 30 mL of acidified methanol (1% HCl + 95% methanol, 15:85 v/v) for 15 min and centrifugation for 3 min at 3000 min^−1^. Finally, the An concentration in the centrifuged extract was measured using a UV/VIS spectrophotometer (UV-2550, Shimadzu, Tokyo, Japan) at 520 nm.

### Selecting top Spectral reflectance indices (SRIs) with two and three bands

**Figure 2:**
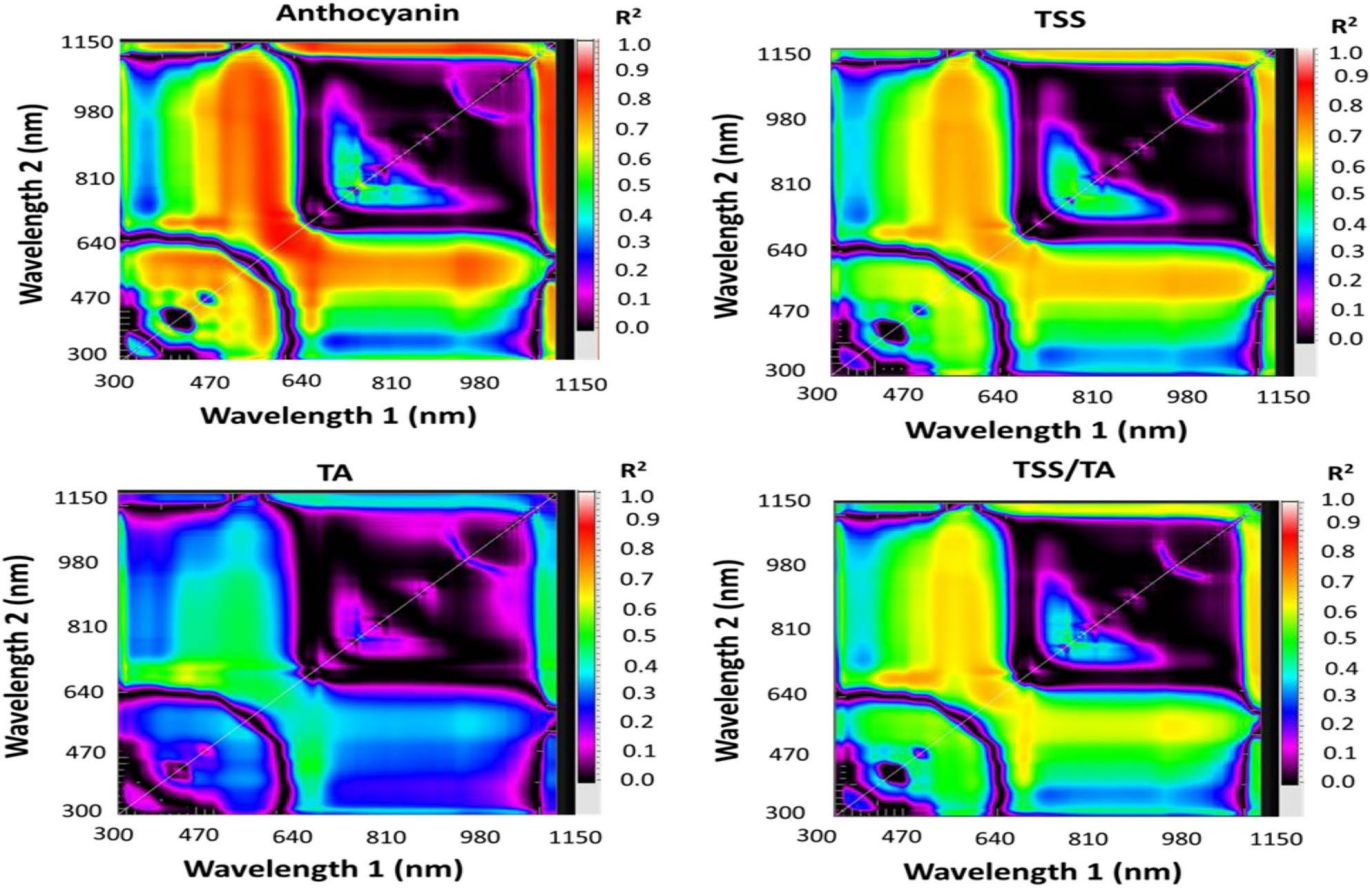
Relationship displaying determination coefficients (R^2^) values for possible dull wavelength together ranging from 302 to 1148 nm with anthocyanin, total soluble solids (TSS), total acidity (TA), and TSS/TA ratio of grape fruits at various ripening stages.

**Figure 3:**
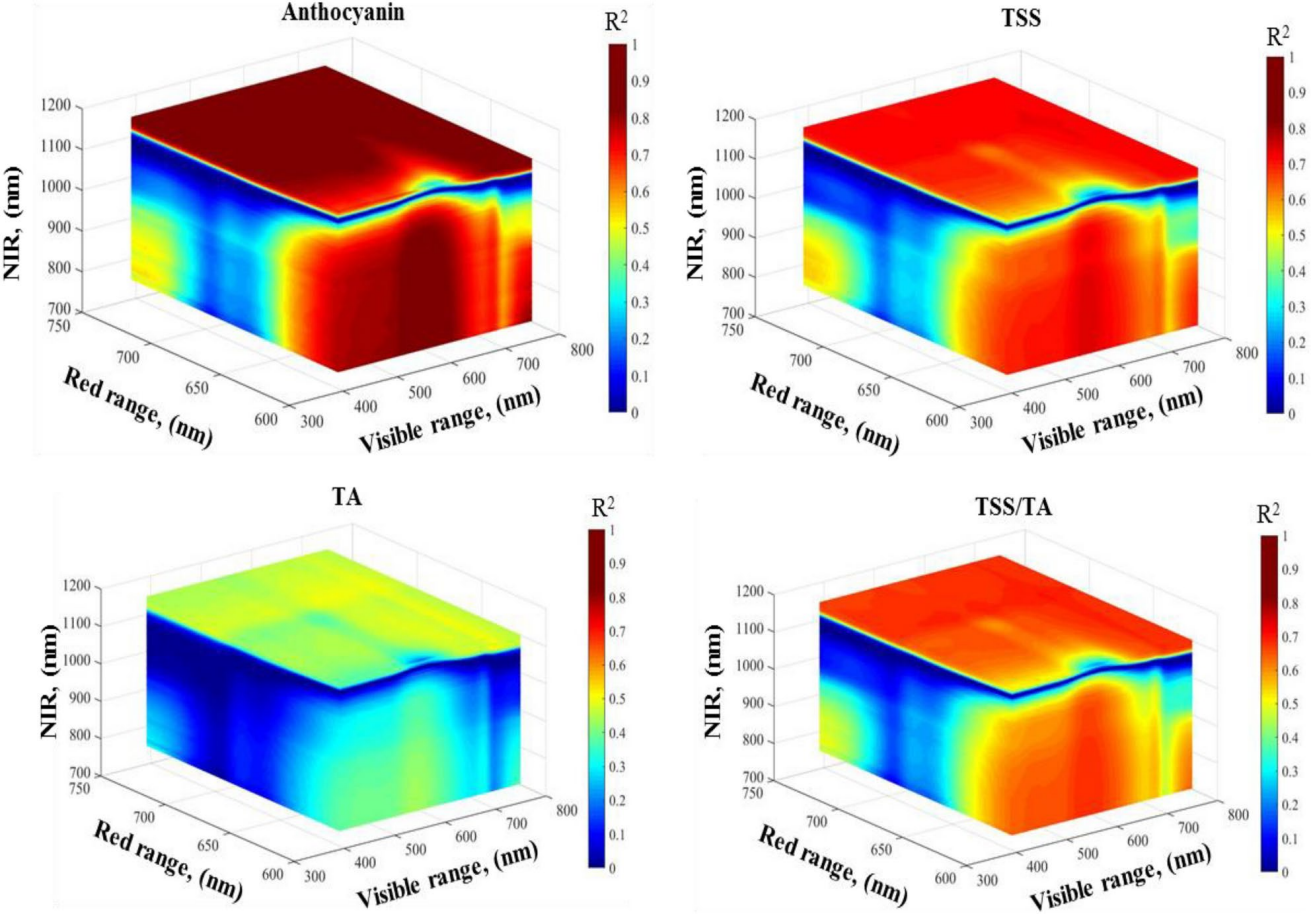
Association matrices display the determination coefficients (R^2^) for all combinations of three spectral bands related to anthocyanin levels, total soluble solids (TSS), total acidity (TA), and the TSS to TA ratio in grapes at various maturity stages.

This study analyzed 30 SRIs, comprising 4 published indices and 26 newly developed ones, categorized into 12 2D-SRIs and 14 3D-SRIs (Table 1). The 2-D (Fig. 2) and 3-D (Fig. 3) contour maps were used to display R^2^ for the relationship between the ripening indicators (An, TSS, TA, and TSS/TA) and all wavelengths within the range of 302–1148 nm. Contour maps (assembled through the MATLAB 2020a software) for each ripening indicator were used to identify the optimal spectral region. These optimized wavelengths are then used to build up the effective SRIs. Based on the R^2^ values, the optimized wavelengths used for building up SRIs were thirty-seven (450, 460, 462, 468, 470, 476, 480, 490, 500, 530, 550, 554, 558, 570, 584, 610, 624, 638, 640, 650, 652, 654, 670, 677, 678, 710, 720, 740, 750, 760, 764, 766, 780, 826, and 970 nm). These wavelengths were used to create the two- and three-band SRIs in both ratio and normalized forms, as shown in Table 1.

**Table 1:** Composition of previously published and recently formulated spectral reflectance indices (SRIs) under investigation within this research

SRIs	Formula	References
Published SRIs		
NDVI	(R_780_ − R_670_)/(R_780_ + R_670_)	Raun et al.^23^
NAI	(R_760_ − R_720_)/ (R_760_ + R_720_)	Rouse et al.^47^
GI	R_554_/ R_677_	Rutkowski et al.^24^
PRMI	(R_750_ − R_678_)/ R_550_	Li et al.^22^
Newly two-band SRIs		
RSI_670,594_	R_670_/R_594_	Present study
RSI_684,1140_	R_684_/R_1140_	
RSI_598,644_	R_598_/R_644_	
RSI_592,650_	R_592_/R_650_	
RSI_610,628_	R_610_/R_628_	
RSI_638,614_	R_638_/R_614_	
RSI_664,434_	R_664_/R_434_	
RSI_668,448_	R_668_/R_448_	
RSI_638,490_	R_638_/R_490_	
RSI_540,950_	R_540_/R_950_	
RSI_626,1018_	R_626_/R_1018_	
RSI_632,964_	R_632_/R_964_	
Newly three-band SRIs		
NDI_822,750,552_	(R_822_ − R_750_ − R_552_)/(R_822_ + R_750_ + R_552_)	Present study
NDI_824,750,554_	(R_824_ − R_750_ − R_554_)/(R_824_ + R_750_ + R_554_)	
NDI_822,750,550_	(R_822_ − R_750_ − R_550_)/(R_822_ + R_750_ + R_550_)	
NDI_1126,696,696_	(R_1126_ − R_696_ − R_696_)/(R_1126_ + R_696_ + R_696_)	
NDI_1126,694,696_	(R_1126_ − R_694_ − R_696_)/(R_1126_ + R_694_ + R_696_)	
NDI_1126,696,694_	(R_1126_ − R_696_ − R_694_)/(R_1126_ + R_696_ + R_694_)	
NDI_1126,698,696_	(R_1126_ − R_698_ − R_696_)/(R_1126_ + R_698_ + R_696_)	
NDI_1126,696,698_	(R_1126_ − R_696_ − R_698_)/(R_1126_ + R_696_ + R_698_)	
NDI_1144,684,562_	(R_1144_ − R_684_ − R_562_)/R_1144_ + R_684_ + R_562_)	
NDI_1144,682,566_	(R_1144_ − R_682_ − R_566_)/R_1144_ + R_682_ + R_566_)	
NDI_1144,682,564_	(R_1144_ − R_682_ − − R_564_)/R_1144_ + R_682_ + R_564_)	
NDI_1148,690,690_	(R_1148_ − R_690_ − R_690_)/R_1148_ + R_690_ + R_690_)	
NDI_1148,688,690_	(R_1148_ − R_688_ − R_690_)/R_1148_ + R_688_ + R_690_)	
NDI_1148,688,688_	(R_1148_ − R_688_ − R_688_)/R_1148_ + R_688_ + R_688_)	

Where RSI and NDI indicate the ratio spectral index and normalized difference index form, respectively. NDVI, NAI, GI, and PRMI indicate normalized difference vegetation index, anthocyanin index, greenness index, and pigment sensitive ripening monitoring index, respectively.

### Machine learning models

#### Gradient boosting regression (GBR)

GBR is a collection of decision trees used for regression or classification purposes. It involves building a series of trees, with each one focusing on predicting the residuals of the previous tree^48^. GBR offers a range of options for hyperparameter tuning and can optimize different loss functions, resulting in a highly adaptable function fit. It can handle both categorical and numerical data without requiring variable pre-processing. It is also cable of effectively handling missing data. To avoid overfitting, basic trees are constructed in each iteration to ensure they can be reliably extrapolated to independent data. Boosting methods consist of three key components: an additive model, weak learners, and a loss function. This approach capable of capturing non-linear relationships, such as wind power curves, by using a variety of differentiable loss functions, and learn naturally between input properties throughout iterations^49^. Gradient boosting machines utilize gradients to identify the limitations of weak models. This is accomplished through an iterative process, with the goal of amalgamating base learners to reduce prediction errors. Decision trees are merged using an additive model, while the loss function is minimized through gradient descent. In the model’s development stage, focus was placed on selecting the appropriate levels for two key factors: the total stages of enhancement to be executed (*Ns*) and the quantity of attributes evaluated for determining the best division (*Mf*). The options for *Ns* included the set (5, 10, 15, 20, 25), while *Mf* could vary among three choices: ‘auto’, ‘sqrt’, or ‘log2’. The process involved fine-tuning these settings to optimize performance, resulting in the establishment of a premier model configured with the optimal combination of these parameters.

#### Decision tree (DT)

Creating decision trees involves training on datasets marked with class labels, referred to as decision tree induction. This method employs a tree-like model akin to a flowchart, featuring various elements such as a root, decision nodes, and leaves. The tree begins at the root node, progresses through decision nodes—which facilitate the transition between nodes—and concludes at the leaf nodes, where the outcomes from the decision-making points are displayed. Certain decision tree algorithms are only capable of producing binary trees, which have exactly two internal nodes, while others have the ability to generate non-binary trees^50^. In the training phase, attention was given to three key elements: the tree’s utmost depth (*Md*), the least number of samples per leaf (*Ms*), and the highest number of leaf nodes (*Mln*). The parameter values for Md, Ms, and Mln were (1, 3, 5, 7, 9), (2, 4, 6, 8, 10), and (none, 10, 20, 30, 40, 50), respectively. The parameter ccp_alpha, representing cost complexity pruning alpha, was fixed at a value of 1 × 10^−6^. During training, hyperparameter optimization was conducted to create the top-level model using the most effective parameter values. Regression rules can be easily derived from decision trees, making them a suitable for exploratory knowledge discovery. Building decision tree models for regression tasks can be achieved without the need for specialized knowledge in the field or setting up parameters, and they efficiently manage data with many variables.

### Model performance optimization framwork

**Figure 4:**
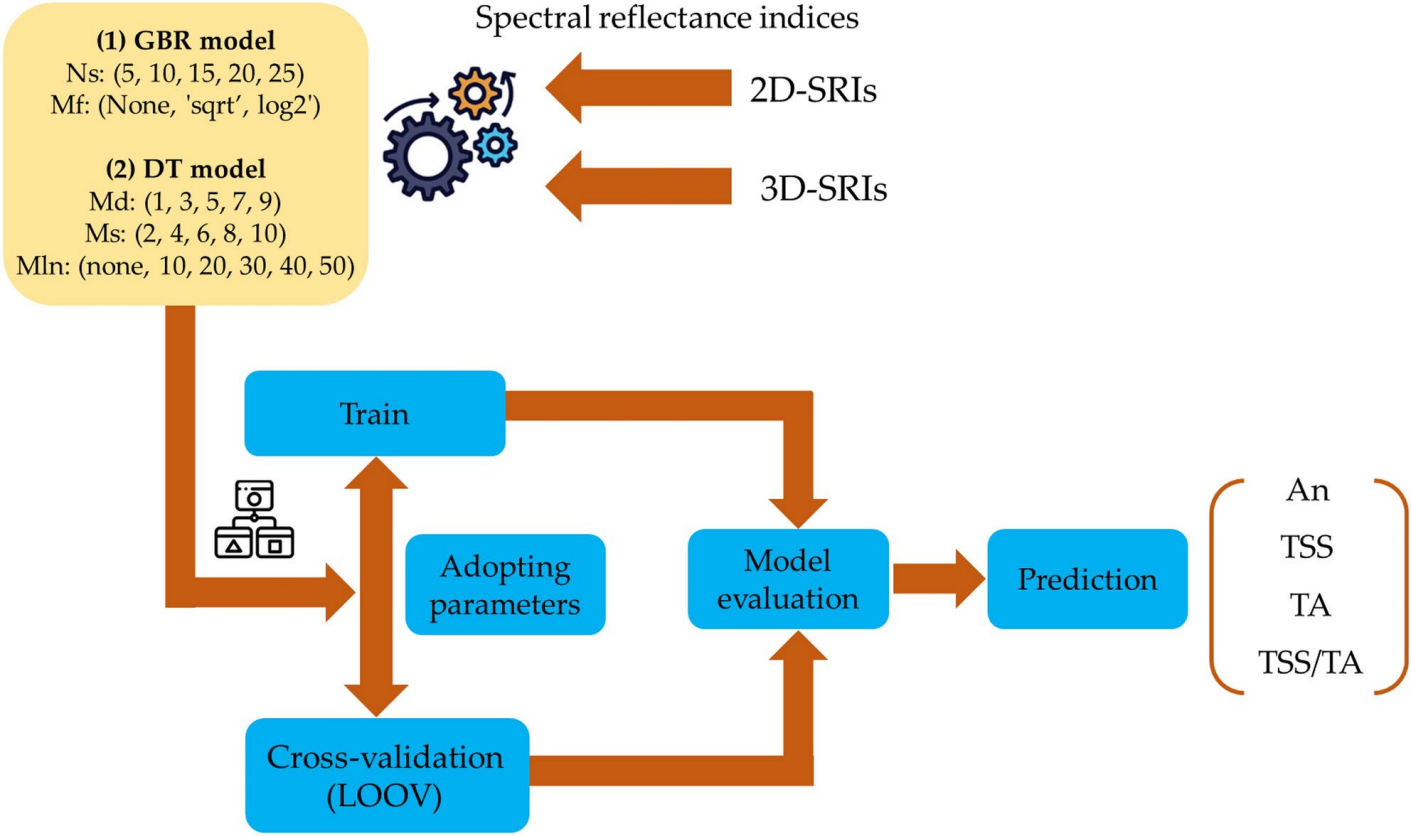
Flowchart depicting the proposed methodology for optimizing model performance.

Figure 4 illustrates the development process for the DT and GBR models, which utilize inputs such as 2D-SRIs and 3D-SRIs to predict parameters including An, TSS, TA, and the TSS/TA ratio. The entire dataset is used for both training and cross-validation (CV). The GBR model is optimized by adjusting hyperparameters like Ns and Mf, while the DT model is fine-tuned using Md, Ms, and Mln. After training, both models undergo Leave-One-Out Cross-Validation (LOOCV) to assess performance, with the objective of minimizing the root mean square error (RMSE). LOOCV is particularly effective for providing an unbiased estimate of model performance, as it tests the model on each individual sample while training on the remaining data^51^. This approach ensures a rigorous evaluation of the model’s generalization ability, making it well-suited for situations with limited data where accurate performance assessment is crucial^52^.

### Tools for data analysis and data splitting

In this work, 105 grape samples were utilized to train and validate the model through LOOCV. In this technique, one sample is left out during each iteration for validation, helping to minimize overfitting and improve the model’s predictive accuracy^53^. All computational tasks, including data processing, model development, and preparation, were performed using Python version 3.12.4. The regression analysis employed the GBR and DT algorithms from the Scikit-learn library, version 1.4.2. The analyses were carried out on a computer with an Intel Core i7-3630QM CPU at 2.4 GHz and 8 GB of RAM.

### Model assessment

Two statistical parameters were used to evaluate the effectiveness of a regression model, including coefficient of determination (R^2^) and the RMSE^54,55^. For this analysis, the variables are specified in the following manner: “Y_act_” represents the value determined through laboratory testing, “Y_p_” is the value forecasted or modeled, “Y_ave_” indicates the mean value, and “T” encompasses the entirety of the data points.

1 $${\text{RMSE }} = \sqrt {\frac{1}{{\text{N}}}\mathop \sum \nolimits_{{{\text{i}} = 1}}^{{\text{T}}} \left( {{\text{Y}}_{{\text{act}}} - {\text{Y}}_{{\text{p}}} } \right)^{2} }$$

2 $${\text{R}}^{2} { = }\frac{{\sum \left( {{\text{Y}}_{{\text{act}}} - {\text{Y}}_{{\text{p}}} } \right)^{2} }}{{\sum \left( {{\text{Y}}_{{\text{act}}} - {\text{Y}}_{{{\text{ave}}}} } \right)^{2} }}$$

### Analysis of statistical data

Variance analysis (ANOVA) was applied to various parameters’ data to assess differences among several maturity categories. The means of each parameter were compared between three ripening groups using Tukey’s test at a significant level of 5%. The box plot was used to present the descriptive statistics of the four parameters. Additionally, Pearson’s correlation matrix was used to analyse the relationship between the four parameters within each ripening group and the pooled data of the three ripening groups. Simple regressions were used to display the relationship between the SRIs and the measured parameters using Sigma Plot 11.0., R^2^ values and significance levels were determined at 0.001.

## Results and discussion

### Variation in ripening indicators among the three ripening groups

**Figure 5:**
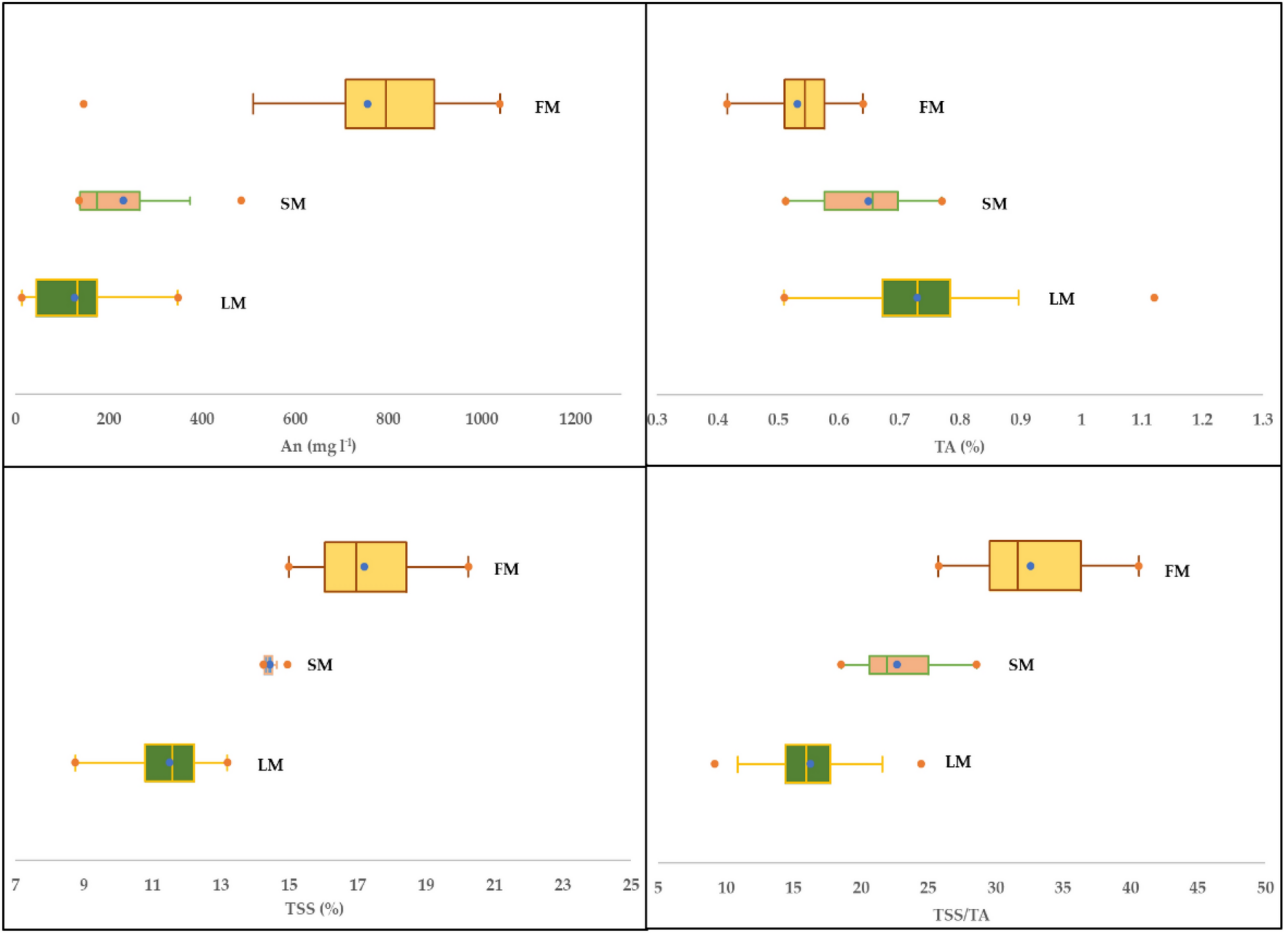
Box plots showing the variation in the values for anthocyanin (An), total soluble solids (TSS), total acidity (TA), and TSS/TA ratio for less mature (LM), semi-mature (SM), and fully mature (FM) ripened grapes.

The descriptive statistics for the four ripening indicators (An, TSS, TA, and TSS/TA) for each ripening group are presented in Fig. 5 (as a box plot) and Table 2. In general, the values of An, TSS, and TSS/TA increased gradually as the fruit ripened, while the opposite trend was observed with TA (Fig. 5 and Table 2). The average values of An, TSS, and TSS/TA for the fully mature (FM) ripened group were 83.3%, 33.1%, and 50.1% higher than those for the less mature (LM) ripened group. Additionally, they were 69.4%, 16.0%, and 30.3% higher than those for the semi-mature (SM) ripened group, respectively. However, the average values of TA for LM were 11.1% and 27.1% higher than those for SM and FM, respectively (Table 2). Furthermore, the three groups of mature grape fruits show a wide range of values for four parameters. The values of An ranged from 13.75 to 1038.75 mg L^−1^, TSS ranged from 8.75 to 20.25%, TA ranged from 0.42 to 1.12%, and TSS/TA ranged from 9.20 to 40.63 (Table 2). Generally, fruit color is usually determined by different pigments such as chlorophylls, carotenoids and anthocyanins^56,57^. The red color of berry skin is a consequence of biosynthesis and accumulation of anthocyanin in the cells, which increases as the fruit ripens^56,58,59^. Anthocyanin accumulation in red grapes begins at the phenological stage of véraison and is regulated by a complex mechanism that is influenced by the plant hormone abscisic acid^60,61^. Generally, at the beginning of veraison, ABA plays a critical role in regulating numerous genes, especially those involved in anthocyanin biosynthesis and their signaling pathway^62^.

**Table 2:** Descriptive statistics (DS) of the anthocyanin (An), total soluble solids (TSS), total acidity (TA), and TSS/TA ratio for less mature (LM), semi-mature (SM), and fully mature (FM) ripened grapes.

Parameters	DS	Ripening groups		
		LM	SM	FM
An (mg L^−1^)	Min	13.75	136.75	145.50
	Max	348.25	483.50	1038.75
	Mean	126.13 c	231.41b	755.66 a
	SD	88.36	130.42	203.95
TSS (%)	Min	8.75	14.25	15.00
	Max	13.20	14.95	20.25
	Mean	11.51 c	14.45 b	17.21 a
	SD	0.98	0.24	1.47
TA (%)	Min	0.51	0.51	0.42
	Max	1.12	0.77	0.64
	Mean	0.73 a	0.65 b	0.53 c
	SD	0.12	0.09	0.05
TSS/TA	Min	9.20	18.56	25.78
	Max	24.51	28.61	40.63
	Mean	16.28 c	22.72 b	32.61 a
	SD	3.49	3.53	4.05

Max, Min, and SD are the maximum, minimum, and standard deviation values, respectively.

The TSS was found to be low for LM group, with a mean value of 11.51% and high for FM group, with a mean value of 17.21%. These findings are consistent with previous studies, which have shown that as fruits ripen, their TSS content increases rapidly in the early stages of ripeness and then slows down as they approach full ripeness^63–66^. This is attributed to the enzymatic breakdown of starch into simple sugars during the ripening process^67,68^. The TA content exhibited an opposite trend to the TSS content, showing higher levels for LM (0.73%) and lower levels for FM (0.53%). Additionally, the TSS/TA ratio increased as TSS levels increased, and TA values decreased throughout the ripening process. This is due to the degradation of organic acids, which often occurs as a result of respiration or conversion to sugars during ripening^69^.

### Relationship between four ripening indicators

**Figure 6:**
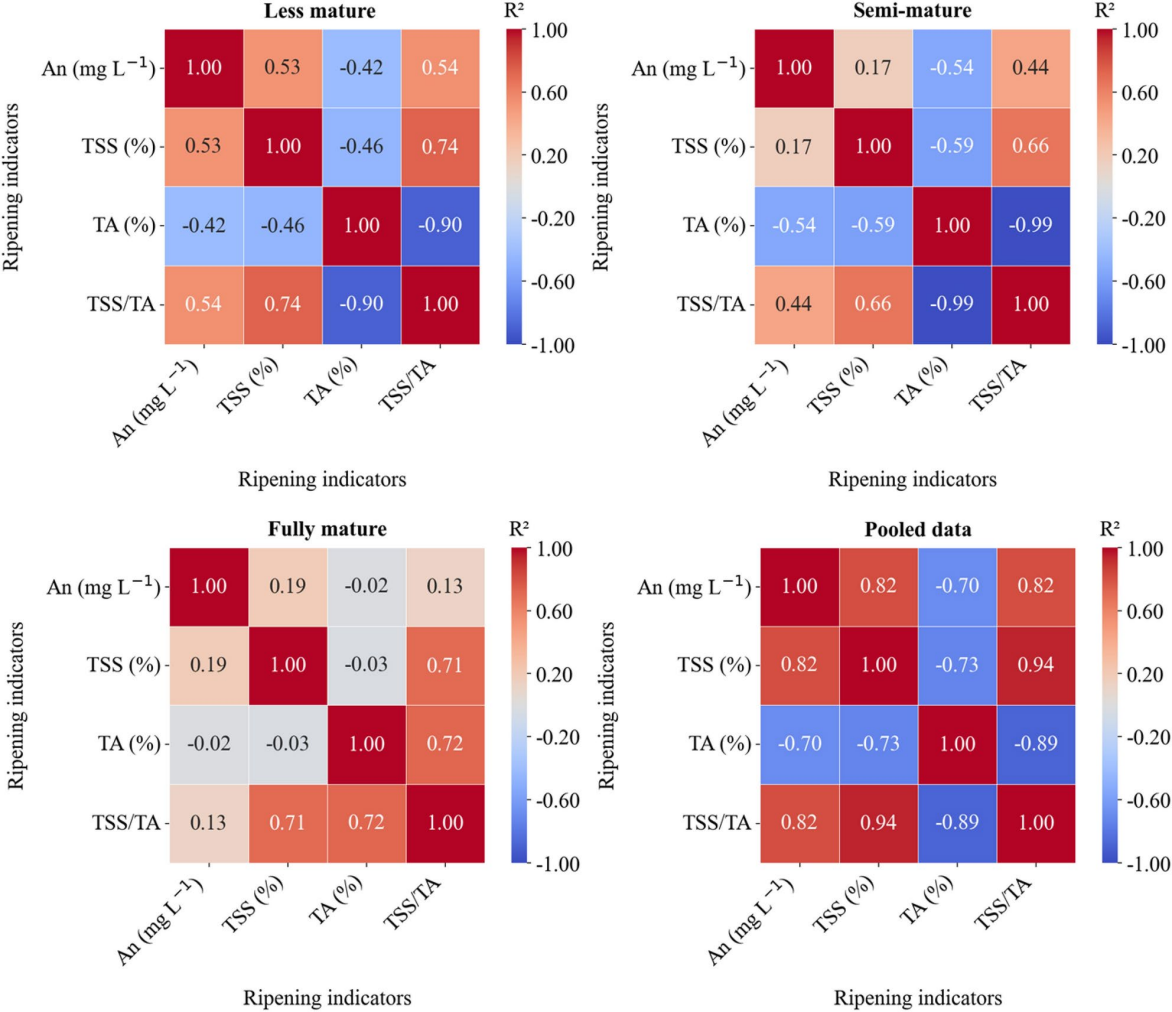
Pearson’s correlation matrix of anthocyanin (An), total soluble solids (TSS), total acidity (TA), and TSS/TA ratio for less mature (LM), semi-mature (SM), and fully mature (FM) ripened grapes as well as for pooled data**.**

When data for the three ripening groups were combined, a strong and positive correlation was observed between An, TSS, and TSS/TA ratio (r = 0.82−0.94). Conversely, TA showed a strong and negative correlation with An (−0.70), TSS (−0.73), and TSS/TA ratio (−0.89) (Fig. 6). For SM and FM groups, the An showed a weak positive and non-significant correlation with TSS. However, this correlation was moderate and significant for the LM group (Fig. 6; derived via Python 3.12.4). For both LM and SM groups, An showed a moderate inverse relationship with TA, while demonstrating a moderate direct relationship with the TSS/TA ratio. However, these correlations were not significant for the FM group. The TSS exhibited a strong positive correlation with the TSS/TA ratio across the three ripening groups. However, it showed a moderate negative correlation with TA for the LM and SM groups but did not exhibit a significant correlation with each other for the FM group (Fig. 6). Wei et al.^66^ found that the TSS and their ratio with TA in grape exhibited a gradual increasing trend of “slow-fast-slow,” while the TA initially increased and then decreased as fruits ripened. This may help to clarify why there is a noticeable and consistent negative correlation between TA and TSS, as well as the TSS/TA ratio, for LM and SM groups. However, there was no longer a significant correlation between AN, TSS, and TA for the FM group. This suggests that the fruits had attained optimal ripeness, characterized by peak levels of An and TSS, and the lowest level of TA.

### Association between spectral reflectance measurements and ripeness metrics

**Figure 7:**
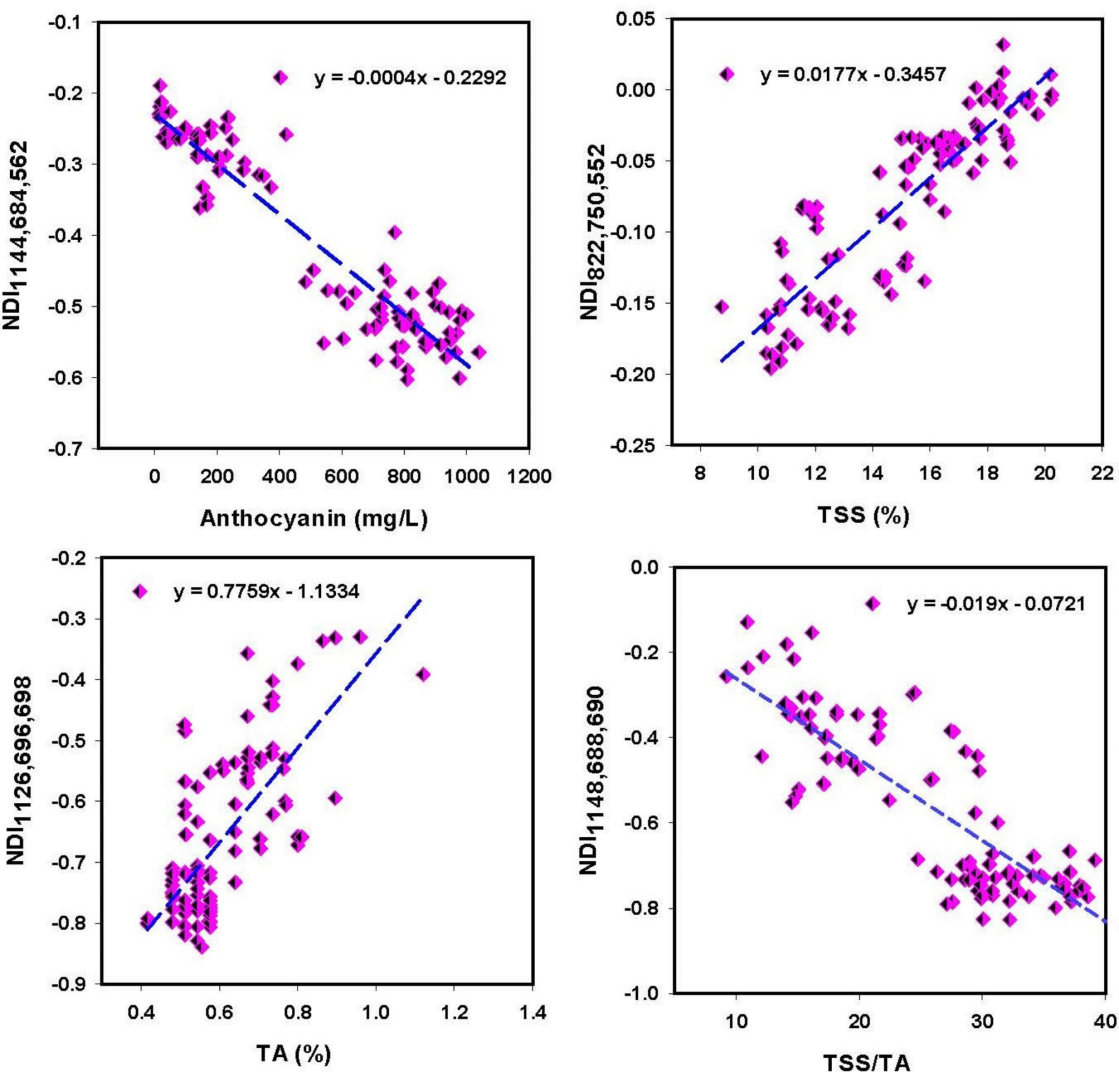
Relationships between spectral reflectance indices and anthocyanin, total soluble solids (TSS), total acidity (TA), and TSS/TA under different ripening stages.

The majority of the tested SRIs demonstrated a significant association with the ripening indicators of grape fruits across three ripening groups, with R^2^ values ranging from 0.19 to 0.88 (Table 3). Strong and significant associations have been found between most assessed SRIs derived from the NIR and VIS wavelengths and different ripening indicators of grape fruits. The newly developed three-band SRIs showed the highest R^2^ with ripening indicators, followed by the newly developed two-band SRIs. For instance, the index NDI_1144,684,562_ and NDI_822,750,552_ showed the highest R^2^ values with An (R^2^ = 0.88), and TSS (R^2^ = 0.76), respectively. The NDI_1126,696,698_ and NDI_1148,688,690_ achieved the highest R^2^ value with TA (R^2^ = 0.51) and TSS/TA (R^2^ = 0.70), respectively (Fig. 7).

**Table 3:** Adjusted coefficients of determination quantify the proportion of variance in anthocyanin (An), total soluble solids (TSS), total acidity (TA), and the TSS/TA ratio explained by various spectral reflectance indices (SRIs) across all collected data.

SRIs	An	TSS	TA	TSS/TA
NDVI	0.00^ns^	0.01^ns^	0.00^ns^	0.00^ns^
NAI	0.25**	0.26**	0.03ns	0.19*
GI	0.71***	0.66***	0.45***	0.62***
PRMI	0.71***	0.66***	0.35***	0.59***
RSI_670,594_	0.84***	0.68***	0.39***	0.64***
RSI_684,1140_	0.84***	0.65***	0.42***	0.63***
RSI_598,644_	0.86***	0.73***	0.46***	0.69***
RSI_592,650_	0.85***	0.72***	0.46***	0.68***
RSI_610,628_	0.87***	0.73***	0.46***	0.69***
RSI_638,614_	0.86***	0.71***	0.42***	0.66***
RSI_664,434_	0.66***	0.58***	0.49***	0.60***
RSI_668,448_	0.70***	0.61***	0.49***	0.62***
RSI_638,490_	0.84***	0.65***	0.42***	0.63***
RSI_540,950_	0.78***	0.71***	0.41***	0.65***
RSI_626,1018_	0.58***	0.47***	0.16*	0.42***
RSI_632,964_	0.57***	0.45***	0.15*	0.40***
NDI_822,750,552_	0.77***	0.76***	0.43***	0.68***
NDI_824,750,554_	0.78***	0.76***	0.43***	0.68***
NDI_822,750,550_	0.77***	0.76***	0.43***	0.68***
NDI_1126,696,696_	0.75***	0.64***	0.51***	0.63***
NDI_1126,694,696_	0.75***	0.65***	0.51***	0.63***
NDI_1126,696,694_	0.75***	0.65***	0.51***	0.63***
NDI_1126,698,696_	0.75***	0.65***	0.51***	0.63***
NDI_1126,696,698_	0.75***	0.65***	0.51***	0.63***
NDI_1144,684,562_	0.88***	0.69***	0.44***	0.67***
NDI_1144,682,566_	0.88***	0.69***	0.44***	0.67***
NDI_1144,682,564_	0.88***	0.69***	0.44***	0.66***
NDI_1148,690,690_	0.84***	0.73***	0.50***	0.70***
NDI_1148,688,690_	0.84***	0.73***	0.50***	0.70***
NDI_1148,688,688_	0.84***	0.73***	0.49***	0.70***

*,**,***Statistically significant at P ≤ 0.05, P ≤ 0.01, and P ≤ 0.001, respectively, ns indicates not significant. NDVI, NAI, GI, and PRMI indicate normalized difference vegetation index, anthocyanin index, greenness index, and pigment sensitive ripening monitoring index, respectively.

A comparative analysis between the newly developed spectral reflectance indices (NDIs) and the existing indices is crucial to demonstrate the advantages of the new indices. Table 3 reveals that the established indices, such as NDVI, NAI, GI, and PRMI, show moderate to strong correlations with key plant traits like TSS and TA. For example, GI and PRMI exhibit very strong correlations with An (R^2^ = 0.71) and TSS (R^2^ = 0.66), indicating their reliability in assessing these traits. Similarly, indices like RSI_670,594_ and RSI_598,644_ consistently show high correlations with An (R^2^ = 0.84) and TSS (R^2^ = 0.73), demonstrating their effectiveness in capturing plant physiological conditions. However, these indices are based on two-wavelength combinations, which may limit their ability to detect the full range of spectral variations associated with complex plant biochemical changes. On the other hand, the newly developed NDI indices, such as NDI_822,750,552_ and NDI_1144,684,562_, show exceptionally high correlations with An (R^2^ = 0.77 and 0.88) and TSS (R^2^ = 0.76 and 0.69), suggesting that the three-wavelength approach in these new indices enhances sensitivity and accuracy. These indices offer improved precision in monitoring biochemical traits, likely due to their ability to better differentiate plant characteristics across a broader spectral range. The comparative results indicate that while the older indices provide reliable data, the newly developed NDIs may offer more nuanced insights into plant biochemistry. For instance, NDI_1148,690,690_ (R^2^= 0.84) and NDI_822,750,550_ (R^2^ = 0.76) show strong correlations with An and TSS, highlighting their potential for capturing finer spectral differences, which are crucial for more accurate assessments of plant health and stress. These findings ultimately highlight the superior accuracy, sensitivity, and capacity of the newly developed indices to detect complex physiological traits in plants.

Most of the selected SRIs were found to be significantly associated with specific ripening indicators of grape fruits. For example, The R^2^ values for An ranged from 0.25 to 0.88, from 0.26 to 0.76 for TSS, from 0.15 to 0.51 for TA, and from 0.19 to 0.70 for TSS/TA. A based on their findings, Pires et al.^70^ recommended using non-destructive methods to assess the ripeness of Citrus by predicting internal quality parameters. These methods showed strong predictive accuracy for TSS (R^2^ = 0.79) and pH (R^2^ = 0.80), as well as for titration acidity (R^2^ = 0.73) and maturity index (R^2^ = 0.80). In their study, Galal et al.^71^ found that the Anthocyanin index (NAI) and R_800/R640_ showed the highest value (R^2^ = 0.89 for both) in relation to chlorophyll content, while the R_570/R540_ index had the greatest value (R^2^ = 0.79) in relation to TSS. In a study by Huang et al.^27^ spectroscopy was used to assess the quality of tomato. The findings revealed that spectroscopy was highly effective in predicting the pH and TSS levels of the tomatoes. Specifically, the study reported a regression coefficient of 0.81 for predicting pH and 0.80 for TSS. In addition, Wati et al.^72^ found that the most accurate model for measuring the pH of intact tomato used a wavelength range of 527–799 nm, resulting in an R^2^ value of 0.90.

The analysis of Tables 3 and 4 reveals notable differences in the performance of SRIs in explaining the variance in grapevine characteristics (An, TSS, TA, and TSS/TA ratio) across various maturity stages: less mature (1st stage), semi-mature (2nd stage), fully mature (3rd stage), as well as in the combined dataset. In Table 3, which combines data from all maturity stages, several indices stand out as highly effective in predicting grapevine traits. In contrast, Table 4, which breaks down R^2^ values by individual maturity stages, shows that certain indices perform better during specific stages. For example, GI excels in the 2nd maturity stage with an R^2^ of 0.74 for An, but its effectiveness drops in the 1st and 3rd stages. RSI_670, 594_ performs well in the 2nd maturity stage for An (R^2^ = 0.77) but is less effective in the 1st and 3rd stages. Similarly, RSI_598, 644_ and RSI_592, 650_ show strong results in the 2nd stage for TSS, but their performance is more variable across the other stages. Likewise, NDI_822,750,552_ and NDI_1148,688,688_ perform well for TSS/TA in the 2nd stage, but their consistency diminishes in other stages. The data fusion of SRIs across all collected data (Table 3) is crucial, as it synthesizes results from all stages, offering a more comprehensive understanding of the spectral reflectance indices’ predictive power. This fusion plays a key role in the accurate prediction and monitoring of grapevine traits, underscoring its potential applications in precision agriculture and grapevine quality assessment.

**Table 4:** Adjusted coefficients of determination for the variance in anthocyanin (An), total soluble solids (TSS), total acidity (TA), and TSS/TA ratio explained by various spectral reflectance indices (SRIs) across three grape maturity stages: less mature (1st stage), semi-mature (2nd stage), and fully mature (3rd stage).

SRIs	An			TSS			TA			TSS/TA		
	1st	2nd	3rd	1st	2nd	3rd	1st	2nd	3rd	1st	2nd	3rd
NDVI	0.06^ns^	0.00^ns^	0.00^ns^	0.25*	0.02^ns^	0.15*	0.01^ns^	0.00^ns^	0.07^ns^	0.07^ns^	0.01^ns^	0.00^ns^
NAI	0.23*	0.61***	0.01^ns^	0.30*	0.48**	0.10*	0.40**	0.30*	0.05^ns^	0.43**	0.45**	0.00^ns^
GI	0.47**	0.74***	0.02^ns^	0.20*	0.26*	0.01^ns^	0.03^ns^	0.23*	0.01^ns^	0.06^ns^	0.29*	0.00^ns^
PRMI	0.44**	0.60***	0.00^ns^	0.06^ns^	0.17*	0.11*	0.00^ns^	0.17*	0.06^ns^	0.00^ns^	0.20*	0.00^ns^
RSI_670,594_	0.21*	0.77***	0.15*	0.08^ns^	0.28*	0.00^ns^	0.01^ns^	0.22*	0.04^ns^	0.00^ns^	0.29*	0.03^ns^
RSI_684,1140_	0.41**	0.70***	0.11*	0.13*	0.20*	0.01^ns^	0.00^ns^	0.19*	0.01^ns^	0.02^ns^	0.23*	0.02^ns^
RSI_598,644_	0.36*	0.81***	0.12*	0.14*	0.30*	0.00^ns^	0.00^ns^	0.24*	0.03^ns^	0.02^ns^	0.32*	0.04^ns^
RSI_592,650_	0.38**	0.81***	0.11*	0.15*	0.30*	0.00^ns^	0.00^ns^	0.24*	0.03^ns^	0.02^ns^	0.32*	0.04^ns^
RSI_610,628_	0.37*	0.80***	0.12*	0.15*	0.29*	0.00^ns^	0.01^ns^	0.24*	0.03^ns^	0.02^ns^	0.32*	0.04^ns^
RSI_638,614_	0.28*	0.78***	0.12*	0.11*	0.30*	0.00^ns^	0.00^ns^	0.23*	0.05^ns^	0.01^ns^	0.31*	0.04^ns^
RSI_664,434_	0.21*	0.43**	0.04^ns^	0.06^ns^	0.17*	0.01^ns^	0.38**	0.14*	0.00^ns^	0.25*	0.19*	0.00^ns^
RSI_668,448_	0.24*	0.48**	0.02^ns^	0.06^ns^	0.19*	0.01^ns^	0.32*	0.16*	0.00^ns^	0.21*	0.21*	0.00^ns^
RSI_638,490_	0.37*	0.71***	0.13*	0.12*	0.20*	0.01^ns^	0.00^ns^	0.20*	0.01^ns^	0.01^ns^	0.23*	0.02^ns^
RSI_540,950_	0.38**	0.75***	0.00^ns^	0.07^ns^	0.35*	0.15*	0.00^ns^	0.29*	0.07^ns^	0.00^ns^	0.36*	0.00^ns^
RSI_626,1018_	0.32*	0.82***	0.03^ns^	0.33*	0.55***	0.09^ns^	0.48**	0.41**	0.07^ns^	0.52***	0.56***	0.01^ns^
RSI_632,964_	0.31*	0.79***	0.03^ns^	0.30*	0.57***	0.07^ns^	0.53***	0.45**	0.08^ns^	0.53***	0.60***	0.02^ns^
NDI_822,750,552_	0.37*	0.79***	0.00^ns^	0.06^ns^	0.38**	0.27*	0.01^ns^	0.27*	0.07^ns^	0.01^ns^	0.37*	0.00^ns^
NDI_824,750,554_	0.37*	0.80***	0.00^ns^	0.06^ns^	0.39**	0.26*	0.01^ns^	0.28*	0.07^ns^	0.01^ns^	0.37*	0.00^ns^
NDI_822,750,550_	0.38**	0.79***	0.00^ns^	0.07^ns^	0.38**	0.27*	0.01^ns^	0.27*	0.07^ns^	0.01^ns^	0.37*	0.00^ns^
NDI_1126,696,696_	0.57***	0.68***	0.07^ns^	0.21*	0.25*	0.13*	0.10*	0.20*	0.00^ns^	0.12*	0.26*	0.03^ns^
NDI_1126,694,696_	0.57***	0.68***	0.07^ns^	0.21*	0.25*	0.13*	0.10*	0.21*	0.00^ns^	0.12*	0.26*	0.03^ns^
NDI_1126,696,694_	0.57***	0.68***	0.07^ns^	0.21*	0.25*	0.13*	0.10*	0.21*	0.00^ns^	0.12*	0.26*	0.03^ns^
NDI_1126,698,696_	0.57***	0.67***	0.07^ns^	0.21*	0.25*	0.12*	0.10*	0.20*	0.00^ns^	0.12*	0.26*	0.03^ns^
NDI_1126,696,698_	0.57***	0.67***	0.07^ns^	0.21*	0.25*	0.12*	0.10*	0.20*	0.00^ns^	0.12*	0.26*	0.03^ns^
NDI_1144,684,562_	0.28*	0.82***	0.16*	0.06^ns^	0.24*	0.00^ns^	0.00^ns^	0.25*	0.03^ns^	0.00^ns^	0.30*	0.04^ns^
NDI_1144,682,566_	0.31*	0.81***	0.17*	0.07^ns^	0.23*	0.00^ns^	0.00^ns^	0.25*	0.03^ns^	0.01^ns^	0.30*	0.04^ns^
NDI_1144,682,564_	0.27*	0.82***	0.17*	0.06^ns^	0.23*	0.00^ns^	0.00^ns^	0.25*	0.03^ns^	0.00^ns^	0.30*	0.04^ns^
NDI_1148,690,690_	0.46**	0.80***	0.09^ns^	0.13*	0.24^*^	0.01^ns^	0.05^ns^	0.25*	0.06^ns^	0.06^ns^	0.30*	0.03^ns^
NDI_1148,688,690_	0.45**	0.80***	0.09^ns^	0.13*	0.24*	0.01^ns^	0.05^ns^	0.25*	0.06^ns^	0.06^ns^	0.31*	0.03^ns^
NDI_1148,688,688_	0.44**	0.80***	0.10*	0.13*	0.24*	0.00^ns^	0.04^ns^	0.25*	0.06^ns^	0.05^ns^	0.31*	0.03^ns^

Where *,**,*** Statistically significant at P ≤ 0.05, P ≤ 0.01, and P ≤ 0.001, respectively, ns indicates not significant.

### Performance of integration SRIs with decision tree and gradient boosting regression models for predicting the ripening indicators

Table 5 shows the integration of two-band SRIs (2D-SRIs) and three-band SRIs (3D-SRIs) with the DT model for filtering high-level variables and accurately predicting the four grape ripening indicators (An, TSS, TA, and TSS/TA ratio). The DT model underwent training with 3D-SRIs and 2D-SRIs to forecast the observed ripening indicators. Subsequently, the DT model’s forecasted values were juxtaposed with the real values. The study’s multivariate analysis and comparative techniques demonstrate a significant improvement in predictability with this approach. LOOCV evaluates model performance by iteratively using each data point as a validation set, averaging error metrics across iterations. While effective for small datasets, it can be computationally intensive for larger ones. The findings of this study reveal that integrating DT with the four hybrid SRIs (HSRIs) achieved superior accuracy in forecasting An. The R^2^ values were 0.93 for the training set and 0.87 for the CV datasets, with corresponding RMSE values of 87.62 and 87.81, respectively. The integration of DT with two HSRIs has demonstrated the highest reliability in forecasting TSS, achieving R^2^ scores of 0.86 and 0.81, along with RMSE values of 1.06 and 0.97 for the training and CV datasets, respectively. Concerning TA, the amalgamation of DT with four HSRIs emerged as the most precise predictor, exhibiting R^2^ values of 0.81 and 0.61, and RMSE values of 0.05 and 0.05 for the training and CV sets, respectively. Furthermore, the model of DT with five HSRIs showcased outstanding performance in predicting TSS/TA, with R^2^ values of 0.89 and 0.74, and RMSE values of 2.71 and 3.12 for the training and CV sets, respectively.

**Table 5:** Outcomes of the decision tree (DT) model based on the data fusion of 2D-SRIs, 3D-SRIs, and Optimal SRIs.

Variable	Proposed features	Optimal parameters	Train		Cross-validation	
		(Md, Ms, MLn)	R^2^	RMSE	R^2^	RMSE
An	All	(3, 10, none)	0.92***	93.74	0.85***	94.32
	RSI_664,434_, GI, RSI_540,950_, NDI_1144,682,566_	(3, 6, none)	0.93***	87.62	0.87***	87.81
TSS	All	(3, 10, 10)	0.87***	1.02	0.73***	1.13
	NDI_822,750,550_, RSI_664,434_	(3, 8, none)	0.86***	1.06	0.81***	0.97
TA	All	(5, 2, none)	0.93***	0.03	0.51***	0.06
	RSI_668,442_, RSI_540,950_, RSI_626,1018_, RSI_664,434_	(5, 4, 10)	0.81***	0.05	0.61***	0.05
TSS/TA	All	(5, 4, 10)	0.89***	2.71	0.69***	3.42
	NDVI, NAI, RSI_664,434_, NDI_822,750,552_, RSI_540,950_	(5, 4, 10)	0.89***	2.71	0.74***	3.12

Where Ms, Md, and Mln represent the minimum samples per leaf, the maximum depth of the tree, and the maximum leaf nodes, respectively. *** indicates statistical significance at P ≤ 0.001.

In Table 6, the utilization of 2D-SRIs and 3D-SRIs in training the GBR model for forecasting four grape ripening indicators is demonstrated. The findings indicate that the GBR model with seven HSRIs demonstrated superior accuracy in predicting An, achieving impressive R^2^ values of 0.95 and 0.86, along with RMSE values of 72.55 and 99.36 for the training and CV sets, respectively. Likewise, the GBR model with ten HSRIs emerged as the best predictor for TSS, delivering R^2^ scores of 0.94 and 0.82 and RMSE scores of 0.70 and 0.92 for the training and CV sets, respectively. In terms of TA, the GBR model with six HSRIs demonstrated the highest accuracy, with RMSE values of 0.04 and 0.05, and R^2^ values of 0.88 and 0.70 for the training and CV sets, respectively. Furthermore, compared to alternative models, the model of GBR with eight HSRIs outperformed in predicting TSS/TA, achieving R^2^ values of 0.93 and 0.77, and RMSE values of 2.09 and 3.17 for the training and CV sets, respectively.

**Table 6:** Outcomes of the gradient boosting regression (GBR) model based on the data fusion of 2D-SRIs, 3D-SRIs, and Optimal SRIs.

Variable	Proposed features	Optimal parameters	Train		Cross-validation	
		(Ns, Mf)	R^2^	RMSE	R^2^	RMSE
An	All	(25, None)	0.96***	63.49	0.84***	106.70
	RSI_670,594_, NDI_1148,690,690_, RSI_592,650_, GI, RSI_638,614_, NDI_1144,684,562_, NDI_1148,688,688_	(25, log2)	0.95***	72.55	0.86***	99.36
TSS	All	(25, log2)	0.94***	0.70	0.82***	0.96
	NDI_1126,696,694_, RSI_540,950_, NDI_1126,694,696_, NDI_824,750,554_, NDI_822,750,550_, NDI_822,750,552_, RSI_668,442_, RSI_610,628_, GI, RSI_664,434_	(25, log2)	0.94***	0.70	0.82***	0.92
TA	All	(20, None)	0.87***	0.04	0.59***	0.05
	RSI_664,434_, RSI_626,1018_, RSI_668,442_, NDI_822,750,550_, RSI_540,950_, NDI_1144,684,562_	(25, sqrt)	0.88***	0.04	0.70***	0.05
TSS/TA	All	(25, None)	0.93***	2.09	0.76***	3.35
	RSI_626,1018_, RSI_670,594_, R_822,750,552_, NAI, RSI_668,442_, RSI_664,434_, R_1144,684,562_, RSI_680,1140_	(25, None)	0.93***	2.09	0.77***	3.17

Where *Ns* represent the count of boosting stages to execute, while *Mf* indicates the number of features to evaluate. *** specifies statistical significance at P ≤ 0.001.

As indicated by studies Elsayed et al.^73^ and Elmetwalli et al.^74^ have demonstrated that the importance of implementing multiple practices, such as refining broad features and fine-tuning model parameters, all aimed at boosting prediction accuracy. These adjustments have led to better-than-expected performance in regression models. Additionally, the outstanding performance of deep learning algorithms in classification can be attributed to four primary factors. These factors include the selection of the most appropriate feature for a color space image, the integrating of image data with environmental information about plants, the implementation of data augmentation, and combination of multiple trained deep networks^75^.

Lastly, this study highlights the significant advantage of integrating spectral indices with machine learning models, demonstrating improved accuracy in predicting grape ripening indicators. By refining features and optimizing parameters, the proposed approach offers a reliable, non-invasive method for monitoring crop development, which can reduce labor and costs while enhancing decision-making in viticulture. Future research could expand on the study findings by incorporating deep learning techniques to further enhance predictive accuracy, utilizing larger datasets and integrating dynamic environmental variables. Additionally, advancements in remote sensing technology and real-time monitoring systems hold the potential to make these methods more efficient and scalable, promoting sustainable practices in agriculture.

## Conclusion

A cost-effective approach was developed to indirectly assess grape ripening indicators by combining spectral reflectance indices (SRIs) with decision tree (DT) and gradient boosting regression (GBR) models. The newly designed three-band SRIs outperformed two-band SRIs, demonstrating superior accuracy in evaluating grape ripening. This was evidenced by their higher R^2^ values when predicting four key ripening indicators: anthocyanin (An), total acidity (TA), total soluble solids (TSS), and the TSS/TA ratio across three ripening stages. Integrating various types of SRIs with DT and GBR models offers a reliable procedure for accurately estimating the four ripening indicators. The analysis outcomes indicated that the three-band spectral reflectance indices (NDIs) showed higher R^2^ values in correlating with grape ripeness indicators, particularly for An and TSS. For instance, NDI_1144,684,562_ attained an R^2^ value of 0.88 in predicting An content, NDI_822,750,552_ achieved an R^2^ value of 0.76 for TSS content, NDI_1126,696,696_ recorded an R^2^ value of 0.51 for TA content, and NDI_1148,690,690_ obtained an R^2^ value of 0.70 for TSS/TA content. Additionally, the newly developed NDIs surpassed conventional indices like GI (R^2^ = 0.71 and 0.66) and PRMI (R^2^ = 0.71 and 0.66) in estimating TSS and An content. However, GI exhibited the highest performance for TA (R^2^ = 0.45) and TSS/TA (R^2^ = 0.62). The use of multiple SRIs significantly enhanced predictive accuracy across all biochemical traits. Notably, the DT model delivered excellent results for An (R^2^ = 0.87, RMSE = 87.81) and TSS/TA (R^2^ = 0.74, RMSE = 3.12). Similarly, the GBR model excelled in predicting TSS (R^2^ = 0.82, RMSE = 0.92) and TA (R^2^ = 0.70, RMSE = 0.05). These results underscore the potential of integrating novel SRIs with advanced machine learning models for precise evaluation of grapevine ripening characteristics. The future research could incorporate additional SRIs, hybrid modeling techniques, and environmental data to improve predictive precision and facilitate real-time applications in sustainable viticulture.

## Data Availability

All data are presented within the article.
